# Patients' Payments and Contributions

**Published:** 1916-12-02

**Authors:** 


### THE MODIFIED PAY SYSTEM.

### Patients' Payments and Contributions.

### II.—A REVIEW OF THE METHODS OF FIFTY HOSPITALS.

Inquiry was made recently at fifty hospitals, where the accounts revealed that payments by patients were received, in order to discover how far and in what direction the contributing idea is progressing to-day. In almost every case it was found that the principle is accepted in one form or another, although not always in respect of in-patients. The methods of contribution may be classified as follows:—

1. An invitation is given to in-patients to make a donation.
2. Fees are paid by patients towards cost of butter and groceries, and in some cases these and other articles of food are provided by the patients.
3. Payment is made according to means by patients, who are mixed in general wards.
4. Payment is made towards cost of dressings and medicines by out-patients.
5. Payment is made according to means in wards graded according to amounts paid.
6. Separate large contributing wards at uniform fees.
7. A paying home apart from the hospital.

Within these separate groups there are variations as to arrangements for medical service and as to special food or extra facilities for visitors, which will be dealt with in turn. For convenience we will refer to each of the groups in the order in which they are arranged above.

### Different Methods in Detail.

1. *Invitation to make a Donation.*—The success of this plan will depend upon the manner in which the invitation is made, and whether it is or is not what an Irishman in certain circumstances called a "cordial invitation." To ask a patient or his friends to give after discharge, when the warm sense of benefit is beginning to fade, is not likely to elicit a large return. In any case the system of invitation to make a voluntary payment, while proving that the justice of a contribution is recognised by the hospital, is unfair to the patients as a whole, because a number who could and should pay escape free.

2. *Fees for Groceries, etc.*—This system is generally considered to be archaic, and as it exists (in very few cases, we believe) clearly means contributing fees in another name. Where in rare instances actual articles of food are provided the system is open to obvious objection, because food is a part of the treatment and should not be of unknown origin.

3. *Payment in Mixed Wards.*—In eighteen of the fifty hospitals investigated, varying payments are taken from patients placed in general wards. These payments are assessed by the house committee, the secretary, the almoner, or (as in one case) by the matron. The chief objection lies in the inconvenience caused by patients comparing notes, becoming dissatisfied, and asking for a revision of rates. However, the justice of varying rates, especially when the assessment has been made carefully, is not open to question, and, in fact, accentuates the charitable nature of the work, for those who are able to give nothing, or very little, benefit by the gifts of the charitable in a measure governed by the individual degree of need. In no case inquired into is there any special privilege, as regards visitors or superior diet or the like, accorded to a patient contributing in this way. Probably many of the hospitals in this group would have graded wards if the nature of the building admitted of that system.

4. *Payments by Out-Patients.*—These, according both to the inquiry referred to and to general knowledge, are fairly common. The patient freely has the advantage of the advice of a medical man of the consultant class, the benefit of the use of expensive equipment and appointments, a share in what is provided by the standing charges of the out-patient department, but pays towards the cost of what he takes away in the shape of medicine or dressings. The rates of payment, if they vary, are generally fixed, within certain limits, by an experienced officer, and in no case is treatment refused because of inability to pay on the part of the patient. It is curious that any hospital, having admitted the justice of these payments, should hesitate to institute also payments by in-patients.

5. *Payments in Graded Wards.*—In twenty-one cases there are separate wards, generally small, in which patients are received, as at the new South London Hospital for Women, at fees according to the accommodation provided. Some of these wards may be described as private rooms in which there is only one bed, and for these the highest fees are paid; in others, where there are more than one patient, a smaller payment is made, and in still larger wards a yet smaller fee. These fees range as follows: £1 1s. (in four cases), £1 11s. 6d. (in one case), £2 2s. (in nine cases), £3 3s. (in nine cases), £4 4s. and £5 5s. (in one case each), and £8 8s. (in one case). In most of the hospitals in this group visitors to these paying wards are afforded extra facilities, and the diet is more liberal than that given in the general wards; the times of meals are in some instances more in accordance with ordinary domestic life than is usually the custom in hospital wards. One hospital arranges late dinner, and another, while providing the same food as in the free wards, cooks the meals for the private rooms in a separate kitchen. In another the food is the same, but the service (plate, china, linen, etc.) is more delicate.

### Medical Service Arrangements.

The arrangements as to medical service differ. In the majority of cases the visiting staff take the patients under their care without remuneration, but this is by no means invariably the practice. In eight hospitals the visiting staff receive fees arranged with the patient. In one case the resident medical officer treats the patients without extra cost to themselves, but if a consultant is required, he is chosen from the visiting staff and receives a fee.

At another institution "approved practitioners" are allowed to treat the patients, and in yet another the invariable rule is that the medical attendant *must* be the patient's own doctor, but should a consultant be required he is chosen from the honorary staff and receives a fee.

6. *Large Contributing Wards with Uniform Fees.*—There are a few hospitals in this group with wards, as described, of from twelve to fifteen beds. The patients, generally speaking, are treated in the same way as those in the general wards, and are under the care of the visiting staff without payment for medical attendance. In one hospital the beds are in cubicles, in another there is a little extra privacy accorded by means of curtains which can be drawn round each bed. These curtains are very useful, as well, for the work of the ward, as they do away with the carrying of screens; they are drawn at night, but for the greater part of the day the patients can see each other and converse as in a general ward. Meal-times are different from those in the free wards. Fees are from £1 1s. to £1 11s. 6d. a week.

7. *Home attached to the Hospital.*—Our sole example is St. Thomas's Hospital, whose Home is alluded to in the first article on this subject. The Home is intended for the use of persons of limited means, and the governors have the right to inquire into the pecuniary circumstances of each patient and the nature of the case before admission. The charge is 12s. a day, and the patients are attended by a resident medical officer without extra fee. There are small rooms for isolation purposes only, or where special circumstances make their use desirable; in these the charge is 24s. a day. The ordinary beds are in cubicles. Patients may, if they choose, place themselves at their own expense under the care of any physician or surgeon in consulting practice, whether attached to the hospital or not. The medical adviser so selected will act in consultation with the resident medical officer, who, further, can himself call in a consultant at the patient's expense should the nature of the case demand it. In cases where operation is advised a necessitous patient can obtain the requisite services free or at small cost, but when able to do so he has to pay the surgeon's ordinary fee.

It may be mentioned that in most of the hospitals described in the above groups, in respect of fixed contributory fees, payments are made in advance and a suitable guarantee is required.

### National Health Insurance and Poor-Law Cases.

There are other patients who in some hospitals make payments themselves or have payments made on their behalf; these are they who are receiving, or expect to receive, allowances under the National Health Insurance Act or the Employers' Liability Acts, or whose maintenance is the responsibility of Poor-Law Guardians. We will deal with these in order.

*National Health Insurance.*—The hospitals for consumption appear to make a uniform charge of 30s. a week for insurance cases, and in some instances the visiting staff receive a special honorarium in respect of the work entailed. The patients are mixed with the ordinary patients, and no special privileges are allowed. In some other hospitals the sickness benefit (7s. 6d. or 10s. a week) of a patient "without dependants" is claimed.

*Employers' Liability.*—Very few hospitals exact a payment because a patient is receiving, or expects to receive, a payment under the Employers' Liability Acts. In one case, where there is usually plenty of time to make the arrangements before the patient is admitted, a payment of 2s. 6d. per day is required where there is an outstanding claim or where an allowance has been made; this payment is generally guaranteed by the insurance company. As hospital treatment is so obviously in the interests of all parties concerned in a claim, the expediency of obtaining it and the justice of paying for it are beyond dispute.

*Poor-Law Cases.*—In several instances Guardians subscribe fairly liberally to the local general hospital and have the privilege of sending suitable cases. In a number of the hospitals inquired into the Guardians pay for each patient from 1s. 10d. to 4s. a day.

### The "Letter" System.

The "letter" system, when it is a live thing in a hospital, must tend to modify any question of payment. The subscriber who uses a "letter" cannot understand how a substantial payment can be demanded in addition from the patient. Fortunately, except in a small group of hospitals, chiefly for consumption, and in some country districts insufficiently served in the hospital sense, "letters" are falling into disuse. It is evident, however, that in some cases the subscriber's recommendation is taken into consideration when a question of payment arises. For instance, we find in one hospital for consumption that the ordinary contributing fee is 25s. a week, but if a "letter" is produced it is only 15s. a week. In another for the same disease the weekly fees are 8s. and 12s. with a "letter," and 15s. without. Another institution requires 5s. a week with a recommendation, and 10s. without.

The hospitals from which our information has been gathered are representative institutions, special and general in character, and situated in London, all parts of the provinces, Scotland, and Ireland. There are, no doubt, many others which would also afford proof that the contributing system is becoming more and more recognised as a means for strengthening the finances of our voluntary hospitals, as an arrangement affording assistance in time of need to a class which is necessitous although not very poor, and as an opportunity to inculcate the principles of self-help and providence in a community which is in danger of relying too much upon chance for entirely gratuitous assistance in every emergency.

*** For the first article see The Hospital, November 18, 1916, page 149.

